# Key clinical beam parameters for nanoparticle-mediated radiation dose amplification

**DOI:** 10.1038/srep34040

**Published:** 2016-09-23

**Authors:** Alexandre Detappe, Sijumon Kunjachan, Pascal Drané, Shady Kotb, Marios Myronakis, Douglas E. Biancur, Thomas Ireland, Matthew Wagar, Francois Lux, Olivier Tillement, Ross Berbeco

**Affiliations:** 1 Department of Radiation Oncology, Dana-Farber Cancer Institute, Brigham and Women’s Hospital, Harvard Medical School, Boston, US; 2 Lyon-1 University, Institut Lumière Matière, CNRS UMR5306, Lyon, France; 3 LA-ICP-MS and ICP-ES Laboratories, Boston University, Boston, MA 02215, US

## Abstract

As nanoparticle solutions move towards human clinical trials in radiation therapy, the influence of key clinical beam parameters on therapeutic efficacy must be considered. In this study, we have investigated the clinical radiation therapy delivery variables that may significantly affect nanoparticle-mediated radiation dose amplification. We found a benefit for situations which increased the proportion of low energy photons in the incident beam. Most notably, “unflattened” photon beams from a clinical linear accelerator results in improved outcomes relative to conventional “flat” beams. This is measured by significant DNA damage, tumor growth suppression, and overall improvement in survival in a pancreatic tumor model. These results, obtained in a clinical setting, clearly demonstrate the influence and importance of radiation therapy parameters that will impact clinical radiation dose amplification with nanoparticles.

Nanoparticles composed of high atomic number materials can amplify the effects of radiation therapy while maintaining current clinical radiation dose constraints on healthy organs^1^,^2^. Radiation dose amplification from metallic nanoparticles occurs when incident photon beams produce short range secondary electrons via the photoelectric effect^3^,^4^,^5^. The cross-section for photoelectric interactions is proportional to Z^4^-Z^5^, where Z is the atomic number of the metallic atom. Subsequent generation of Auger electrons and reactive oxygen species (ROS) can also enhance the radiation effects^6^,^7^,^8^,^9^. The concept of nanoparticle-mediated radiation dose amplification has been demonstrated in preclinical models for several nanoparticle formulations^10^,^11^,^12^,^13^ mainly in low energy (keV) preclinical photon beams^10^,^11^,^14^,^15^,^16^,^17^,^18^. However, the translational significance of these studies are limited as low energy photons have poor tissue penetration and are seldom used clinically^19^. The current study is focused on the evaluation of clinical radiation beam parameters for dose amplification. We evaluated the impact of treatment field size, distance from the central beam axis, tumor depth in tissue, and “flattening filter free” (FFF) delivery on nanoparticle-mediated radiation dose amplification.

Most often, high energy photon beams (≥6 MV) generated by a medical linear accelerator are used to treat cancer due to the increased skin sparing properties. However, these high energy beams are actually composed of a spectrum of photon energies, including a substantial component of low energy photons (<150 kV)^20^,^21^. In additon, scatter within the medium/tissue contributes to an increasing proportion of low energy photons as a function of depth. Preliminary investigations of nanoparticle radiation dose amplification in clinical photon beams have been performed^22^,^23^,^24^,^25^,^26^,^27^. We present the first investigation of key clinical parameters that must be considered prior to clinical translation.

In modern radiation therapy, medical linear accelerators generate high energy electron beams which are directed to a Cu/W target to produce photons for therapy. This photon beam is shaped by several different methods depending on the clinical application. Collimator jaws and/or smaller multi-leaf collimators (MLC) give the radiation beam a size and shape conforming to the shape of the therapy target (*e.g*. tumor). A flattening filter is often used to create a homogeneous radiation dose across the treatment field within the patient. This filter also removes many low energy photons and effectively reduces the overall dose-rate^27^,^28^. The medical linear accelerators are mounted on a rotating gantry and patients are placed on robotic treatment “couches” enabling radiation delivery from multiple angles to avoid healthy organs-at-risk. Depending on the angle of radiation delivery, some amount of healthy tissue must be traversed by the photon beam before reaching the radiotherapy target. Three-dimensional radiation dose calculations are performed using analytic algorithms or Monte Carlo methods for clinical treatment planning purposes. During the planning process, several treatment parameters can be optimized in order to maximize the dose to the tumor while minimizing dose to healthy tissue. For this experimental study, we have used a pancreatic adenocarcinoma model. Pancreatic cancers have a low survival rate^20^ and are difficult to treat effectively with radiation due to their anatomical location, as well as inter-fractional and intra-fractional position uncertainties. Recent clinical evidence also suggests that pancreatic tumors may respond to radiation dose escalation^21^. The need for increased efficacy of radiation therapy combined with more precise tumor localization suggests the implementation of a novel agent which can facilitate both radiation dose amplification and increased image contrast, simultanouesly. The nanoparticle tested in this study exhibits high MRI contrast^30^, low toxicity^31^, and high radiation dose amplification^15^,^17^,^32^. AGuIX (Active Guidance for Irradiation by X-rays) are ultrasmall (<5 nm) polysiloxane based nanoparticles with chelates of gadolinium entrapped in a DOTA structure^15^,^17^,^29^,^30^,^31^,^32^,^33^. AGuIX is curently approved for a Phase I clinical trial for magnetic resonance-guided radiation therapy^15^,^19^,^31^,^34^,^35^.

## Results

### Clinical radiation delivery parameters affect *in vitro* radiation dose amplification

**Figure 1:**
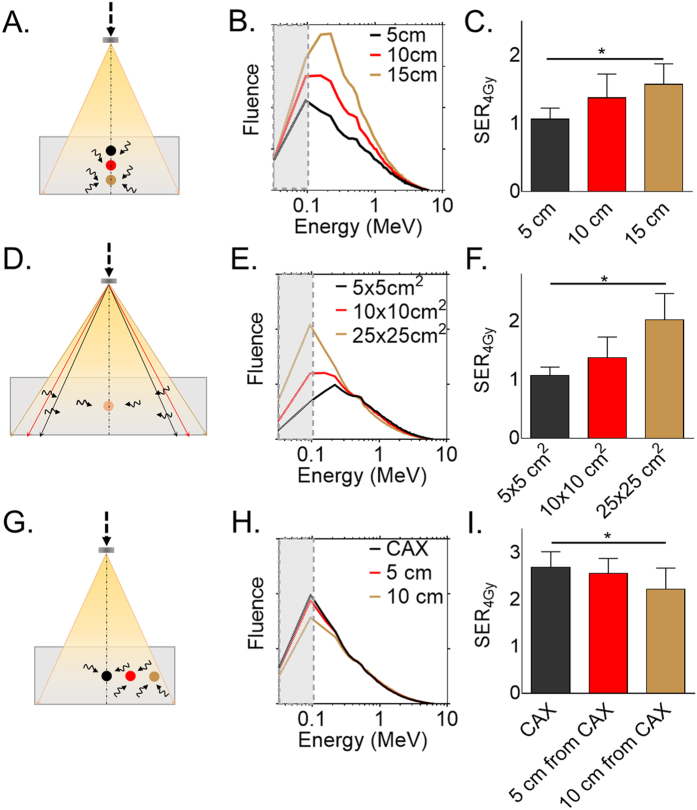
Key clinical beam parameters for nanoparticle-mediated radiation dose amplification. (**A**) Schematic representation of the irradiation (IR) setup for the depth dependence study. The dark arrows represent the expected low energy photons generated by scatter. (**B**) Monte-Carlo simulation of the depth conditions indicate an increasing proportion of low energy photons for greater depths. The grey shaded area shows the range from 10 keV to 100 keV which will interact most strongly with the gadolinium nanoparticles. (**C**) Sensitivity enhancement ratio at 4 Gy (SER_4Gy_) calculated as the ratio of cell survival with IR alone and IR + GdNP. Increased efficacy is shown as a function of depth. Similar experiments were performed by (**D–F**) modifying the field size or (**G–I**) the distance from the central axis (CAX) of the radiation beam. Data are represented as a mean ± SD. Statistical tests were performed using Kruskal Wallis test, * P < 0.05.

The influence of clinical radiation delivery parameters was tested *in vitro* with capan-1 pancreatic adenocarcinoma cells incubated with 0.43 mg/L of silica-based gadolinium nanoparticles (GdNP) before irradiation with a clinical 6 MV photon beam (TrueBeam, Varian Medical Systems, Inc.) (Supplementary Fig. 1). Measurements were made at three depths (5 cm, 10 cm and 15 cm) in solid water (CIRS, Inc) (Fig. 1A). The Monte Carlo-based photon energy spectra (Fig. 1B) shows the increase in the proportion of low energy photons as a function of depth in tissue. We observed a significant increase in sensitivity enhancement ratio at 4 Gy (SER_4Gy_) ranging from 1.01 at 5 cm to 1.48 at 15 cm depth (p < 0.05) (Fig. 1C). Similarly, enlarging the field size also inceases the proportion of low energy photons due to increased scatter within the solid water, leading to greater SER (SER_4Gy_ = 1.01 with 5 × 5 cm^2^ to 1.82 with a 25 × 25 cm^2^ field size, p < 0.05) (Fig. 1D–F). Moving the point of measurement laterally from the central axis (CAX) to the edge of the treatment field decreases the proportion of low energy photons leading to lower effect farther from the CAX. We found SER_4Gy_ = 1.82 at the CAX and 1.32 at 10 cm lateral distance from the axis (p < 0.05) (Fig. 1G–I). These results support our hypothesis that clinical conditions that create softer radiation beams (more low energy photons) will improve the dose amplification properties of high Z nanoparticles.

### *In vitro* evaluation of flattening filter free radiation delivery

**Figure 2:**
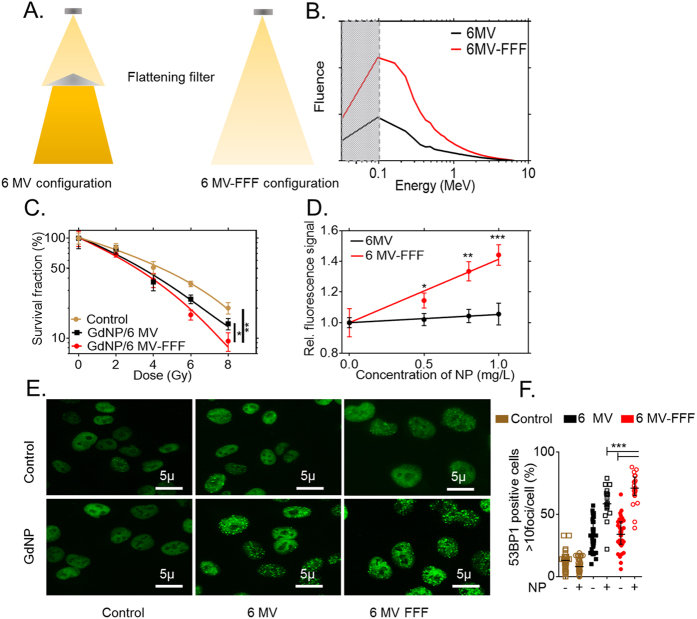
*In vitro* radiation dose amplification studies in clinical 6 MV and 6 MV-FFF radiation beams. (**A**) Schematic representation of the 6 MV and 6 MV-FFF treatment configuration. The flattening filter creates a beam hardening effect. (**B**) The amount of low energy photons is higher for 6 MV-FFF compared to 6 MV, as determined by Monte Carlo simulations. (**C**) Clonogenic survival assay performed with radiation alone shows the increased efficacy of GdNP and 6 MV and 6 MV-FFF. (**D**) Reactive oxygen species measurement quantified as the ratio of fluoresence with and without different doses of GdNP after 4 Gy irradiation. The 6 MV-FFF beam provides greater relative signal at all nanoparticle doses. (**E**) Qualitative and (**F**) quantitative representation of the DNA repair (53BP1) after administration of GdNP and irradiation with 6 MV or 6 MV-FFF after 4 Gy irradiation. Magnification 63x. All data are represented as a mean ± SD. Statistical tests were performed using Kruskal Wallis test, *P < 0.05, **P < 0.01, ***P < 0.001.

Removal of the flattening filter results in higher dose-rate radiation beams with a larger intensity closer to the central axis and more low energy photons overall (Fig. 2A). In Monte Carlo simulations, the 6 MV-FFF beam has 2.6 times as many low energy photons (<100 keV) as the 6 MV beam. (Fig. 2B). Without nanoparticles present, the clonogenic cell survival was not significantly different for the 6 MV-FFF beam compared to the 6 MV beam (Supplementary Fig. S2). After incubation with GdNP, clonogenic cell survival was significantly decreased with the 6 MV-FFF beam compared to the 6 MV beam (P = 0.029) (Fig. 2C). The dose enhancement factor (DEF) obtained with the 6 MV-FFF beam (DEF = 1.36) is similar to the one measured with a preclinical radiation beam (220 kV) (DEF = 1.37)^19^ and is substantially higher than with the 6 MV radiation beam (DEF = 1.22). When combined with GdNP (0.43 mg/L), the 6 MV-FFF beam led to greater ROS generation than 6 MV (P < 0.05). The ROS signal is linear with the radiation dose (R^2^ = 0.97) and the difference between the 6 MV and 6 MV-FFF beams is more significant at higher nanoparticle doses (Fig. 2D). An increase in DNA double strand breaks is observed as suggested by the increase of 53BP1 foci formation induced post-irradiation (Fig. 2E). More than 70% of the cells exhibited 53BP1 foci for the +GdNP/6 MV-FFF group compared to 58% for +GdNP/6 MV (P = 0.0041) (Fig. 2F). Note that the observed density of 53BP1 foci in the control groups are due to the basal DNA-damage of capan-1 cells (approx. 7%)^36^.

### Gadolinium-based nanoparticles biodistribution

**Figure 3:**
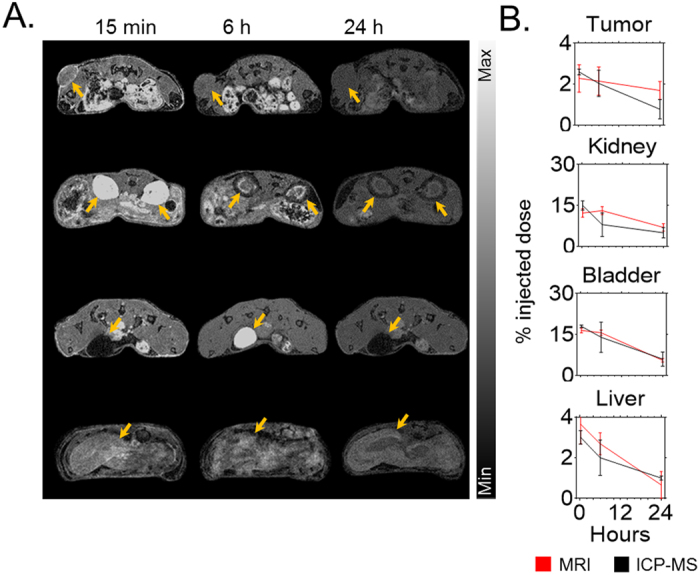
Biodistribution study. (**A**) Axial magnetic resonance images post-i.v. injection of GdNP. Yellow arrows show the tumor, kidney, bladder, and liver uptake of nanoparticles, respectively, between 15 min and 24 h post-injection. (**B**) Biodistribution comparison between non-invasive MRI quantification (n = 3) and ICP-MS (n = 3/time point) after intravenous injection of 0.25 mg/g GdNP.

*In vivo* experiments were carried out using capan-1 tumor-bearing mice. The time point for highest tumor uptake was determined by whole body-MR imaging (T1 map, 7T, Bruker BioSpin, United States) and inductively coupled plasma mass spectrometry (ICP-MS) after systemic injection of 0.25 mg/g of GdNP (Fig. 3A). The nanoparticles were quickly cleared by the kidneys and bladder with a maximum peak in each organ 15 min post injection (15%ID and 18%ID, respectively). After 24 hrs, the GdNP accumulation in the kidneys and bladder were 4%ID and 6%ID, respectively. Due to the lack of lymphatic drainage and the leakiness of the tumor model, a peak in nanoparticle uptake is observed in the MRI data 15 minutes post-injection (~2.3%ID). The ICP-MS measurement confirmed this measurement (2.6%ID) (Fig. 3B).

### *In vivo* therapy studies

**Figure 4:**
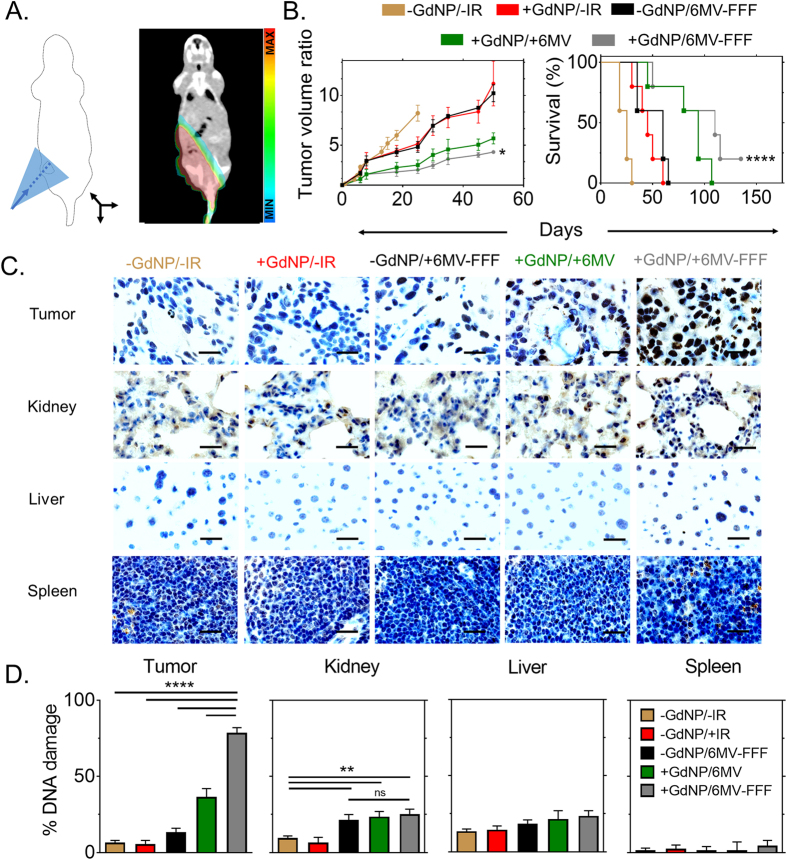
*In vivo* therapy outcomes and toxicity assessment. (**A**) (left) Experimental setup for irradiation with a clinical photon beam and (right) 3D radiation dose calculation using a CT scan and the clinical treatment planning system (**B**) Tumor growth study (n = 5/group) shows a 1.5-fold difference in the tumor size by day 50 between +GdNP/6 MV-FFF and +GdNP/6 MV groups (P = 0.0411) while an extended median lifetime by 16 days (P < 0.0001) was observed in the survival study. (**C**) DNA double strand breaks in the tumor and surrounding tissues shown by γH2AX staining. Scale bar = 20 μm. (**D**) Quantification of γH2AX positive nuclei (‘brown’) counted over 30 images. All data are represented as a mean ± SD. Statistical tests were performed using a Kruskal-Wallis test for the tumor growth study and the DNA damage quantification whereas a Log-Rank test was performed for the survival study. *P < 0.05, **P < 0.01, ***P < 0.001.

6 MV and 6 MV-FFF clinical radiation beams were delivered to capan-1 subcutaneous tumor-bearing mice (Fig. 4A). The tumor growth and survival studies demonstrated a statistically significant benefit for both 6 MV and 6 MV-FFF with GdNP. An approximately 1.5-fold difference in the tumor size by day 50 between +GdNP/6 MV-FFF and +GdNP/6 MV groups (P = 0.0411) was observed and the median lifetime extended by 16 days (+18%) (P < 0.0001) (Fig. 4B). Histopathological evaluation by γΗ2ΑX staining revealed a significant increase in DNA damage for treatment groups that included radiation and GdNP, consistent with the tumor growth and survival results. The rate of DNA damage for 6 MV-FFF/GdNP, 6 MV/GdNP and 6 MV (no GdNP) was 78 ± 4%, 36 ± 6% and 13 ± 3%, respectively) (Fig. 4C,D). For the ipsilateral kidney, a significant increase in DNA damage is observed (P = 0.0019) compared to the non-irradiated groups, indicating a need to ensure kidney sparing is prioritized during the treatment planning process. However, the kidney damage was not significantly greater with nanoparticles present. No other toxicities were observed in any of the other healthy organs studied.

## Discussion

A complete theory of the mechanism responsible for the observed biological effect of nanoparticle-mediated radiation dose amplification is not yet known. The results of this study demonstrate a dependence on low energy photons that supports the hypothesis that the photoelectric effect plays an important role. However, previous studies have suggested that other processes may also contribute to the observed results^3^,^9^,^10^,^37^. We found that situations with increased photoelectric interaction probability (more low energy photons) tend to increase ROS generation as well. Tertiary products, such as hydrogen peroxide, have a longer range of action (a few mm) than photoelectrons (a few μm), increasing the potential to damage the DNA and cause cell death. Recently, Taggart *et al*. demonstrated that protein disulphide isomerase and mitochondrial oxidation could be novel targets for radiosensitization^9^. A full understanding of all of the relevent biophysical factors will be important for designing a nanoparticle strategy that maximizes the therapeutic benefit.

We have shown that key clinical beam parameters can be exploited to increase the efficacy of nanoparticles in external beam radiation therapy. In general, delivering more low energy photons (a softer beam) will result in greater biological effect. The flattening filter free (FFF) delivery mode is a recent clinical innovation mainly used to increase the dose rate and thus decrease the radiation delivery time. This is particularly important in the context of stereotactic treatments in which large amounts of radiation are delivered in a single treatment. That the FFF mode includes a larger proportion of low energy photons is a collatoral advantage for nanoparticle-mediated radiation dose amplification. In the presence of GdNP, FFF beams lead to greater DNA damage and improved survival compared to standard 6 MV beams. Similarly, other technologies such as the modification of the linear accelerator target, as suggested by Berbeco *et al*. could also provide a greater benefit^4^. In that study, it was shown that, by replacing the Cu/W target with a carbon target, the proportion of low energy photons would almost triple at 10 cm depth. In the current study, we show that even a ~40% increase in the low energy photon content has a statistically signifcant effect on tumor growth and survival.

Beyond the radiation beam characteristics, there are other clinical factors which will affect nanoparticle-mediated radiation dose amplification. Tumor vascularity, permeability, and other biological factors will have consequences for nanoparticle uptake, distribution and therapeutic efficacy. Tumor location, visibility on imaging and motion due to respiration or other physiological processes are common clinical challenges which can be alleviated by contrast agents such as the one presented in this study. This is particularly relevant for MR-guided radiation therapy either in the pre-treatment or in-treatment setting. Both are current and emerging modalities in clinical radiation therapy, indicating a growing need for agents that can serve as both MRI contast agents and radiation dose amplification agents. In this context, the AGuIX nanoparticle is uniquely suited to simultaneously provide both greater accuracy and efficacy in clinical radiation therapy.

## Conclusion

We have shown that clinical radiation delivery parameters will have a significant effect on the radiation dose amplification provided by high-Z nanoparticles. Most notable is the benefit of the flattening filter free delivery mode, a common modality for modern radiation therapy procedures. Further advances, both in nanoparticle synthesis and radiation therapy delivery, should provide additional therapeutic advantages.

## Material and Methods

### Monte Carlo simulation

The clinical radiation therapy beams, both 6 MVand 6 MV-FFF were simulated using the Geant4 Monte Carlo code. All beams simulated represent a specific configuration of the linear accelerator which has been experimentally validated in our clinic^38^. A photon fluence is obtained from these simulations for each energy bin between 0 MeV and 6.33 MeV. The simulation model replicated the geometry of our experimental setup: A 6-well plate was placed between two solid water phantoms with lateral dimensions of 30 × 30 cm^2^ and thicknesses of 15 cm (top) and 5 cm (bottom).

### Cell culture

Capan-1 human pancreatic adenocarcinoma cells were acquired from American Type Culture Collection (ATCC, Manassas, VA) and cultured in Iscove’s Modified Eagle Medium, with 20% fetal bovine serum, and 2% Penicillin Streptomycin Glutamine. The cells were stored in a humidified incubator at 37 °C and 5% CO_2_.

### Silica-based gadolinium nanoparticle

Nanoparticles (GdNP) were purchased from CheMatech in their lyophylize form (CheMatech, Dijon, France). For use, the GdNP were resuspended in ultrapure distilled water (Invitrogen, NY) at a concentration of 100 mg/mL before dilution at the appropriate concentration for experiments. The complete physical characterization of these nanoparticles was performed by Lux *et al*.^39^.

### Clonogenic Assay

Capan-1 cells were incubated for 15 minutes with 0.43 mg/mL of GdNP prior to irradiation. Irradiations were performed with either a 6 MV or 6 MV-FFF beam, 90 cm source-to-surface distance (SSD), 10 cm depth in solid water, 15 × 1 cm^2^ field size. Radiation doses of 0, 2, 4, 6, and 8 Gy were used. After irradiation, the cells were allowed to grow for 10 days, before staining with a 1% crystal violet and 10% ethanol dye solution. Measurements were performed in triplicate. The effect of the GdNP is quantified by the calculation of the dose enhancement factor (DEF) using Matlab (V. R2013b). The DEF is the ratio of the area under the survival curves with and without nanoparticles.

### Sensitivity measurements

Capan-1 cells were incubated for 15 minutes with 0.43 mg/mL of GdNP prior to irradiation. Comparison between different irradiation setups were performed to investigate the impact of the change in each clinical parameter. The depths studied were 5 cm, 10 cm and 20 cm and the field sizes were 5 × 5 cm^2^, 10 × 10 cm^2^, and 25 × 25 cm^2^, with a constant source-to-cells distance of 100 cm and a single irradiation to 4 Gy with the 6 MV clinical beam. Sensitivity enhancement ratio at 4 Gy (SER_4Gy_) is defined as the ratio of cell survival with and without nanoparticles at 4 Gy, while the dose enhancement factor (DEF) is defined as the ratio of cell survival with and without nanoparticles from 0 to 8 Gy.

### Reactive oxygen species measurement

Capan-1 cells (10,000 cells/well) were seeded in 96-well plates and grown for 24 h. The cells were then incubated with different concentrations of nanoparticles for 30 min, and washed with PBS to remove nanoparticles that were not internalized by the cells. Afterwards, cells were incubated with 10 μM dihydrorhodamine 123 (DHR123) for 3 h. Prior to irradiation, cells were washed with PBS to remove excess DHR. Irradiations were performed with a single fraction of 4 Gy irradiation (10 cm depth, 15 × 15 cm^2^ field size) with 6 MV or 6 MV-FFF radiation beams. The fluorescence signal was measured 3 h post-irradiation using a plate reader (POLARstar omega, BMG LABTECH) with an excitation wavelength of 480 nm and an emission wavelength of 520 nm.

### DNA damage quantification

Capan-1 cells were irradiated with clinical 6 MV and 6 MV-FFF beams, with and without GdNP (0.43 mg/ml). Immunofluorescence was performed as previously described^40^. Cells were fixed, permeabilized, and incubated with 53BP1 primary antibody (H-300, Santacruz, USA) and secondary antibody (Alexa Fluor IgG 488 goat anti-rabbit) prior to mounting with Dapi Fluoromount-G (SouthernBiotech, USA). Fluorescence microscopy images were analyzed using a Zeiss Axio microscope at 63X magnification. DNA damage induced by the GdNP was determined by counting the number of cells with more than 10 foci.

### *In vivo* experiments

All animal studies were approved and carried out according to the Animal Care and Use Committee of the Dana-Farber Cancer Institute. Immuno-compromised CrTac: NCr-*Fox1nu* mice were injected with 5 × 10^6^ Capan-1 cells subcutaneously in the flank. Tumors were allowed to reach 5 mm in the longest axis before experiment, and a maximum size of 2 cm in the longest axis before euthanasia.

### Biodistribution study

MRI and inductively coupled plasma mass spectrometry (ICP-MS) were used to determine the biodistribution of the nanoparticles in the capan-1 tumor-bearing mice (n = 3). The *in vivo* biodistribution measurements were performed with a dose equivalent of 0.25 mg/g of GdNP injected intravenously. MRI quantification was performed at different time points post injection from 1 min to 24 h by using a T1 RARE-VTR sequence with a repetition time of 9000 ms, echo time of 19.6 ms, and a flip angle of 180°. The acquisition matrix size and reconstructed matrix were 400 pixels × 200 pixels, with a field of view of 200 × 200 μm^2^, and a 3 mm slice thickness. The T_1_ map acquisition was then correlated to the calibration curve to calculate the absolute quantification of nanoparticles. For ICP-MS, animals were sacrificed at 15 min, 6 h, and 24 h post-injection. The organs were dissolved in HCl, HNO_3_ and H_2_O_2_. Gadolinium concentrations were analyzed on a VG Plasma Quad Excell ICP-MS with the isotope Gd^155^.

### Radiation therapy irradiation protocol

A CT scan was performed in order to delineate the tumor and calculate the 3D radiation dose distribution. Animals were anesthetized with a mix of ketamyne/xelazyne (2:1) and wrapped with 2 cm of flexible tissue-quivalent material. The clinical treatment planning system Eclipse (Aria, V.11) was used to calculate the dose distribution in the tumor and healthy organs using the analytical anisotropic algorithm (AAA) for a 5.5 × 10 cm^2^ field size, gantry at 180 degree, and SSD of 90 cm. Blocking of healthy organs and tissue was performed with the primary collimator. A 10 cm depth for the tumor was created with solid water (CIRS, Inc). Dose calculation was performed for the standard and flattening filter free 6 MV irradiation beams. Irradiations were performed 15 mins after intravenous injection of 0.25 mg/g GdNP.

### Survival study

Five groups (-GdNP/-IR; +GdNP/-IR; -GdNP/+6 MV-FFF; +GdNP/+6 MV; +GdNP/+6 MV-FFF) of five mice each were used to measure the therapeutic efficacy. Irradiations were performed 15 minutes after intravenous administration. The tumor response was measured by volume studies using cone-beam CT (CBCT) imaging (65 kV and 0.5 mA). Two CBCT per week were performed after treatment, and the tumor volumes were normalized to the first CBCT acquired before the treatment. Volume was measured using the 3D Slicer software (V. 4.3.1). Animals were euthanized when the tumor size reached 2 cm in the longest axis. Body weight was measured and behavior observed throughout the experiment to assess systemic toxicity.

### DNA damage assessment

Animals were irradiated 15 minutes after intravenous injection of the GdNP following the same procedure as the survival study above. The tumor was harvested 30 min after irradiation and fixed in 2% formalin followed by paraffin embedding. Tumor slices of approximatively 5 μm were cut. Immunohistochemistry was performed with γH2AX staining (antibody Abcam ab11174) as a marker for DNA damage. Images were analyzed using a Zeiss Axio microscope at 63X magnification.

## Additional Information

**How to cite this article**: Detappe, A. *et al*. Key clinical beam parameters for nanoparticle-mediated radiation dose amplification. *Sci. Rep.* **6**, 34040; doi: 10.1038/srep34040 (2016).

## Supplementary Material

Supplementary Information

## References

[b1] Retif P. . Nanoparticles for Radiation Therapy Enhancement: the Key Parameters. Theranostics 5, 1030–1044, 10.7150/thno.11642 (2015). 26155318 PMC4493540

[b2] Thakor A. S. & Gambhir S. S. Nanooncology: the future of cancer diagnosis and therapy. CA: a cancer journal for clinicians 63, 395–418, 10.3322/caac.21199 (2013). 24114523

[b3] McMahon S. J., Paganetti H. & Prise K. M. Optimising element choice for nanoparticle radiosensitisers. Nanoscale 8, 581–589, 10.1039/c5nr07089a (2015). 26645621

[b4] Berbeco R. I. . Low Z target switching to increase tumor endothelial cell dose enhancement during gold nanoparticle-aided radiation therapy. Medical physics 43, 436, 10.1118/1.4938410 (2016). 26745936 PMC4698122

[b5] Lechtman E. . Implications on clinical scenario of gold nanoparticle radiosensitization in regards to photon energy, nanoparticle size, concentration and location. Physics in medicine and biology 56, 4631–4647, 10.1088/0031-9155/56/15/001 (2011). 21734337

[b6] McMahon S. J. . Biological consequences of nanoscale energy deposition near irradiated heavy atom nanoparticles. Scientific reports 1, 18, 10.1038/srep00018 (2011). 22355537 PMC3216506

[b7] Seo S. J. . Enhanced production of reactive oxygen species by gadolinium oxide nanoparticles under core-inner-shell excitation by proton or monochromatic X-ray irradiation: implication of the contribution from the interatomic de-excitation-mediated nanoradiator effect to dose enhancement. Radiation and environmental biophysics 54, 423–431, 10.1007/s00411-015-0612-7 (2015). 26242374

[b8] Ghaemi B. . Harnessing the Cancer Radiation Therapy by Lanthanide-Doped Zinc Oxide Based Theranostic Nanoparticles. ACS applied materials & interfaces 8, 3123–3134, 10.1021/acsami.5b10056 (2016). 26771200

[b9] Taggart L. E. . Protein disulphide isomerase as a target for nanoparticle-mediated sensitisation of cancer cells to radiation. Nanotechnology 27, 215101, 10.1088/0957-4484/27/21/215101 (2016). 27080849

[b10] Kunjachan S. . Nanoparticle Mediated Tumor Vascular Disruption: A Novel Strategy in Radiation Therapy. Nano letters 15, 7488–7496, 10.1021/acs.nanolett.5b03073 (2015). 26418302 PMC5507193

[b11] Maggiorella L. . Nanoscale radiotherapy with hafnium oxide nanoparticles. Future oncology 8, 1167–1181, 10.2217/fon.12.96 (2012). 23030491

[b12] Liu P. . Silver nanoparticles: a novel radiation sensitizer for glioma? Nanoscale 5, 11829–11836, 10.1039/c3nr01351k (2013). 24126539

[b13] Bianchi A. . Targeting and *in vivo* imaging of non-small-cell lung cancer using nebulized multimodal contrast agents. Proceedings of the National Academy of Sciences of the United States of America 111, 9247–9252, 10.1073/pnas.1402196111 (2014). 24927562 PMC4078830

[b14] Hainfeld J. F., Slatkin D. N. & Smilowitz H. M. The use of gold nanoparticles to enhance radiotherapy in mice. Physics in medicine and biology 49, N309–N315 (2004). 15509078 10.1088/0031-9155/49/18/n03

[b15] Kotb S. . Gadolinium-Based Nanoparticles and Radiation Therapy for Multiple Brain Melanoma Metastases: Proof of Concept before Phase I Trial. Theranostics 6, 418–427, 10.7150/thno.14018 (2016). 26909115 PMC4737727

[b16] Bobyk L. . Photoactivation of gold nanoparticles for glioma treatment. Nanomedicine: nanotechnology, biology, and medicine 9, 1089–1097, 10.1016/j.nano.2013.04.007 (2013). 23643529

[b17] Le Duc G. . Toward an Image-Guided Microbeam Radiation Therapy Using Gadolinium-Based Nanoparticles. ACS Nano 5, 9566–9574, 10.1021/nn202797h (2011). 22040385

[b18] Chang M. Y. . Increased apoptotic potential and dose-enhancing effect of gold nanoparticles in combination with single-dose clinical electron beams on tumor-bearing mice. Cancer science 99, 1479–1484, 10.1111/j.1349-7006.2008.00827.x (2008). 18410403 PMC11158140

[b19] Detappe A. . AGuIX nanoparticles as a promising platform for image-guided radiation therapy. Cancer nanotechnology 6, 4, 10.1186/s12645-015-0012-3 (2015). 26345984 PMC4556741

[b20] Miller K. D. . Cancer treatment and survivorship statistics, 2016. CA: a cancer journal for clinicians, 10.3322/caac.21349 (2016). 27253694

[b21] Krishnan S. . Focal Radiation Therapy Dose Escalation Improves Overall Survival in Locally Advanced Pancreatic Cancer Patients Receiving Induction Chemotherapy and Consolidative Chemoradiation. International journal of radiation oncology, biology, physics 94, 755–765, 10.1016/j.ijrobp.2015.12.003 (2016). PMC4792191 26972648

[b22] Liu C. J. . Enhancement of cell radiation sensitivity by pegylated gold nanoparticles. Physics in medicine and biology 55, 931–945, 10.1088/0031-9155/55/4/002 (2010). 20090183

[b23] Liu J., Liang Y., Liu T., Li D. & Yang X. Anti-EGFR-Conjugated Hollow Gold Nanospheres Enhance Radiocytotoxic Targeting of Cervical Cancer at Megavoltage Radiation Energies. Nanoscale research letters 10, 218, 10.1186/s11671-015-0923-2 (2015). 25995714 PMC4437992

[b24] Burger N. . A method for the efficient cellular uptake and retention of small modified gold nanoparticles for the radiosensitization of cells. Nanomedicine: nanotechnology, biology, and medicine 10, 1365–1373, 10.1016/j.nano.2014.03.011 (2014). 24674970

[b25] Berbeco R. I. . DNA damage enhancement from gold nanoparticles for clinical MV photon beams. Radiation research 178, 604–608, 10.1667/RR3001.1 (2012). 23148509 PMC3525114

[b26] Wolfe T. . Targeted gold nanoparticles enhance sensitization of prostate tumors to megavoltage radiation therapy *in vivo*. Nanomedicine: nanotechnology, biology, and medicine 11, 1277–1283, 10.1016/j.nano.2014.12.016 (2015). PMC4476911 25652893

[b27] Detappe A. . Advanced multimodal nanoparticles delay tumor progression with clinical radiation therapy. J Control Release. 238, 103–13, 10.1016/j.jconrel.2016.07.021 (Sep 28, 2016). 27423325

[b28] Yan Y. . Dosimetric differences in flattened and flattening filter-free beam treatment plans. Journal of medical physics / Association of Medical Physicists of India 41, 92–99, 10.4103/0971-6203.181636 (2016). 27217620 PMC4871009

[b29] De Puysseleyr A., Lechner W., De Neve W., Georg D. & De Wagter C. Absorbed dose measurements in the build-up region of flattened versus unflattened megavoltage photon beams. Zeitschrift fur medizinische Physik 26, 177–183, 10.1016/j.zemedi.2016.02.005 (2016). 27020966

[b30] Fries P. . Evaluation of a Gadolinium-Based Nanoparticle (AGuIX) for Contrast-Enhanced MRI of the Liver in a Rat Model of Hepatic Colorectal Cancer Metastases at 9.4 Tesla. RoFo: Fortschritte auf dem Gebiete der Rontgenstrahlen und der Nuklearmedizin 187, 1108–1115, 10.1055/s-0035-1553500 (2015). 26361379

[b31] Sancey L. . Long-term *in vivo* clearance of gadolinium-based AGuIX nanoparticles and their biocompatibility after systemic injection. ACS nano 9, 2477–2488, 10.1021/acsnano.5b00552 (2015). 25703068

[b32] Sancey L. . The use of theranostic gadolinium-based nanoprobes to improve radiotherapy efficacy. The British journal of radiology 87, 20140134, 10.1259/bjr.20140134 (2014). 24990037 PMC4453146

[b33] Mignot A. . A top-down synthesis route to ultrasmall multifunctional Gd-based silica nanoparticles for theranostic applications. Chemistry 19, 6122–6136, 10.1002/chem.201203003 (2013). 23512788

[b34] Luchette M., Korideck H., Makrigiorgos M., Tillement O. & Berbeco R. Radiation dose enhancement of gadolinium-based AGuIX nanoparticles on HeLa cells. Nanomedicine: nanotechnology, biology, and medicine 10, 1751–1755, 10.1016/j.nano.2014.06.004 (2014). 24941464

[b35] Lux F. . Gadolinium-based nanoparticles for theranostic MRI-radiosensitization. Nanomedicine (Lond) 10, 1801–1815, 10.2217/nnm.15.30 (2015). 25715316

[b36] Li Y. H. . Inhibition of non-homologous end joining repair impairs pancreatic cancer growth and enhances radiation response. PloS one 7, e39588, 10.1371/journal.pone.0039588 (2012). 22724027 PMC3377637

[b37] Her S., Jaffray D. A. & Allen C. Gold nanoparticles for applications in cancer radiotherapy: Mechanisms and recent advancements. Advanced drug delivery reviews, 10.1016/j.addr.2015.12.012 (2015). 26712711

[b38] Tsiamas P. . A modification of flattening filter free linac for IMRT. Medical physics 38, 2342–2352, 10.1118/1.3571419 (2011). 21776768

[b39] Lux F. . Ultrasmall rigid particles as multimodal probes for medical applications. Angewandte Chemie 50, 12299–12303, 10.1002/anie.201104104 (2011). 22057640

[b40] Lee D. H. . A PP4 phosphatase complex dephosphorylates RPA2 to facilitate DNA repair via homologous recombination. Nature structural & molecular biology 17, 365–372, 10.1038/nsmb.1769 (2010). PMC3057140 20154705

