# Supplementary material for: Key clinical beam parameters for nanoparticle-mediated radiation dose amplification
Source: Sci Rep. 2016 Sep 23;6:34040. doi: 10.1038/srep34040 (PMC5034311; doi:10.1038/srep34040)
Supplement: Supplementary Information [file srep34040-s1.pdf]

SUPPLEMENTARY INFORMATION

Key clinical beam parameters for nanoparticle-mediated radiation dose amplification

Alexandre Detappe[1,2], Sijumon Kunjachan[1], Pascal Drané[1], Shady Kotb[1, 2], Marios Myronakis[1], Douglas E Biancur[1], Thomas Ireland[3], Matthew Wagar[1], Francois Lux[2], Olivier Tillement[2], Ross Berbeco[1,*]

1. Department of Radiation Oncology, Dana-Farber Cancer Institute, Brigham and Women's Hospital, Harvard Medical School, Boston, US
2. Lyon-1 University, Institut Lumière Matière, CNRS UMR5306, Lyon, France
3. LA-ICP-MS and ICP-ES Laboratories, Boston University , Boston, MA 02215, US

*Corresponding authors:

Ross Berbeco, PhD, DABR

Radiation Oncology

Dana-Farber Cancer Institute, Brigham and Women's Hospital, Harvard Medical School

450 Brookline Avenue, Boston, MA 02215, US

Phone: +1 617-525-7136

E-mail: RBerbeco@LROC.Harvard.edu

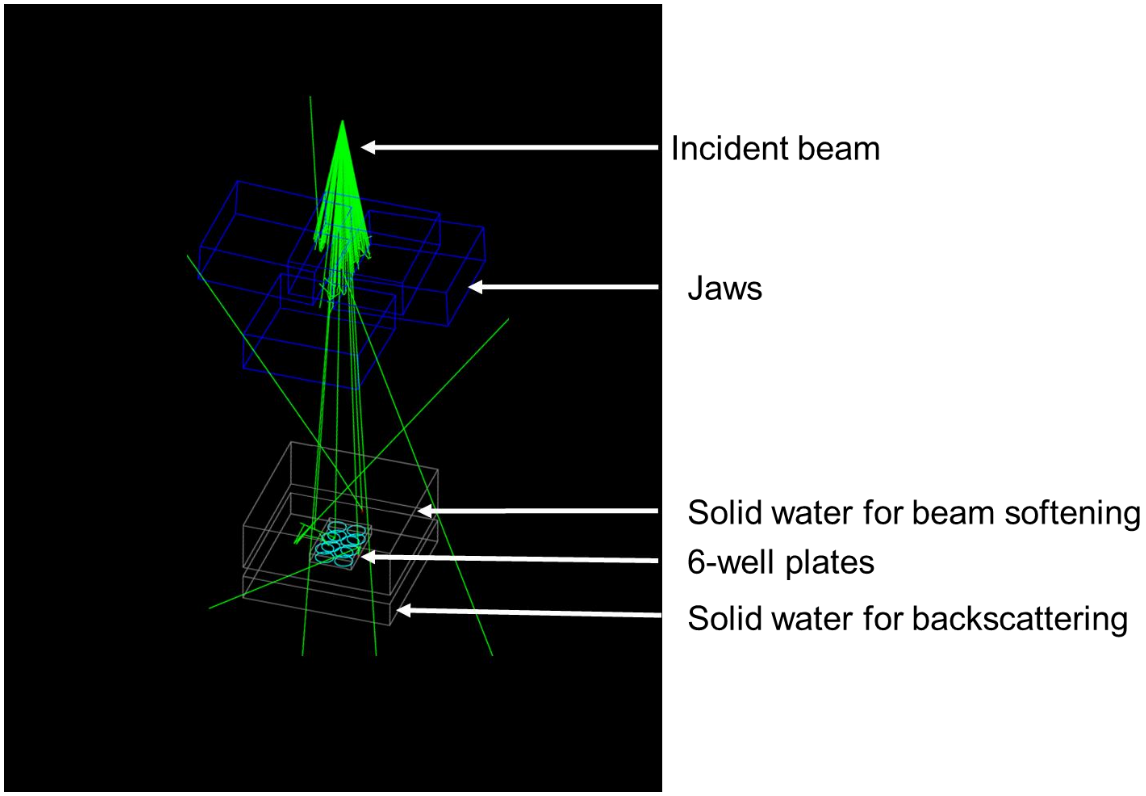

Figure Supplementary 1. Geometry used for the Monte-Carlo simulation of the *in vitro* experiments.

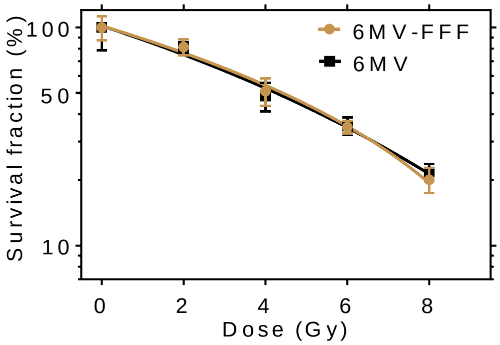

Figure Supplementary 2. No difference in the clonogenic survival is observed between 6 MV and 6 MV-FFF at 10 cm depth, SAD = 100 cm, field size = 15 x 15 cm^2 (no GdNP).
